# On the Question of Excision in Tubercular Hip Disease

**Published:** 1891-11-21

**Authors:** 


### SURGERY.

### ON THE QUESTION OF EXCISION IN TUBERCULAR HIP DISEASE.

In the older days of surgery the hip joint was excised—just as the limb was amputated for diseased knee-joint—because the patient's strength was failing from continued hectic and prolonged suppuration. But of late years it has become the custom with many surgeons to excise the hip in tubercular disease at a much earlier stage, and as an alternative to prolonged rest and drainage of the abscesses in connection with it.

A child with hip disease is treated with a Thomas splint or kept in bed with extension on, and yet the disease progresses, and an abscess forms in connection with the joint. What are we to do in such a case? Are we to continue to treat the case with rest and possibly an antiseptic evacuation of the pus and drainage, or are we to excise the joint in the hope of thereby cutting short the disease?

In trying to answer this question we must satisfy ourselves on the following points: 1. Does excision, at the stage when abscesses form, lessen the mortality? 2. Does it cut short the disease? 3. Does it give a more useful limb than the expectant treatment?

How many cases die when they have progressed to suppuration? This question can be answered from Mr. Marsh's statistics of 172 cases prepared for the Clinical Society some years ago. He showed that about half the cases in which suppuration has occurred, die;* and that in cases with suppuration ending in cure, the average duration of treatment is about four years.

Mr. Croft ten years ago brought before the Clinical Society 45 cases in which he had excised the hip, and, out of these, 18 recovered and 16 died, the result in the remaining cases being uncertain. In only two of the 18 cured cases were the sinuses chronic, and in only six others had the sinuses been opened with persiste e of discharge, so that the majority were not advanced cases; whereas in 11 out of 16 patients who died after excision chronic sinuses were present when excision was performed. The inference seems to be that had these cases been excised earlier the result might have been different, but, at any rate, the excisions were performed so late in many cases that they do not serve to answer our inquiry as to the value of excision at an early stage taken as a whole, though the fact that so many recovered when the joint was excised early certainly points to this as the right treatment.

It was from Mr. Marsh's and Mr. Croft's cases, and some others collected from various hospitals, that the Clinical Society drew up their report on the value of excision in hip disease, and they found the mortality after excision, and in cases treated by rest and extension, almost equal, and they did not recommend the operation. But these were evidently nearly all advanced cases of hip disease, and so they cannot answer our enquiry.

Mr. G. Wright, of the Pendlebury Children's Hospital, has performed excision at the stage of which we write in 100 cases. Of these, 85 recovered and 15 died. This looks very encouraging; but we are told that in 64 cases sinuses remained. Even, however, if we take only the cured and fatal cases, it gives 21 of the former against 15 of the latter. Mr. Wright, whose experience of hip disease is very large, expresses the opinion that excision at this stage tends both to the preservation of life and the cutting short of the disease. But this is not the universal opinion of surgeons, and the question seems to be still *sub judice.* Mr. Bowlby last autumn stated that he did not consider excision necessary, even in suppurating cases. He thought it was enough to open the abscesses, without removing the tuberculous bone.

But all this refers to cases of hip excision, where the wound has been afterwards drained and no attempt made to remove all the diseased tissues of the joint. Last November, at a meeting of the Medico-Chirurgical Society, Mr. Barker brought forward a series of cases of hip excision in which he removed, as far as possible, all the diseased tissue by scraping and cutting it away. This was done so thoroughly that it was possible to close up the wound without drainage, and to get primary union and an early restoration of the use of the limb. All sinuses were thus obviated, and after a few months of rest on a Thomas splint, the disease could be said to be at an end, and the patient able to walk about again.

Surely here we have an excision of the desirability of which we can have little doubt, once suppuration has occurred.

We should always wait for suppuration in these cases, because statistics show that in cases that have not suppurated a large proportion recover with rest, whereas in cases where suppuration has take n place half the cases die. Mr. Barker advises excision as soon as caseation takes place, but the

* We omit all reference to Mr. Marsh's cases in which the result was at all uncertain.

question is can we diagnose this until abscess forms, in the case of the hip joint?

There is another reason for excising the joint to which Mr. Barker has called attention in his lectures on tubercular joint disease, and that is the liability to general infection from the tubercular focus. But this danger is present in cases before suppuration occurs, and certainly often has occurred after excision, so that the infective nature of tubercular joint disease does not seem to us an argument for excision, as we cannot excise every tubercular joint as we should a limb with sarcoma—the infective nature of tubercle is not marked enough.

And now to consider our third question, *i.e.*, as to the condition of the limb. Here again we find opinions differ. Mr. Wright considers it gives a more useful limb than rest and drainage. Mr. Holmes, on the other hand, says "he has never seen any result after operation so perfect as occurred after natural recovery, even when in the latter cases the disease had proceeded to an extreme condition."

After excision, the union is, as a rule, such as to allow of some movement varying much in degree. It is very rarely bony union. The extreme shortening sometimes observed is very often due to the child leaving off the splint too soon and bearing weight on the limb too early, so that the end of the bone gets *forced* up on the dorsum ilii.

On the condition of the limb after excision, as compared with the treatment by rest and extension, the evidence of the committee of the Clinical Society is of great value. They report that "although movement is more frequently present and also more extensive after excision, the patients often walk insecurely, with considerable limp; whilst the limbs after treatment by rest and extension, though frequently more or less fixed, is firmer and more useful for purposes of progression."

To return, then, to our supposed case of hip disease in the suppurating stage. Are we to drain the abscess and trust to rest to complete a cure? Looking at Mr. Marsh's statistics, surely the complete excision of Barker hold out a much better chance for the child. The disease is cut short, and the risks of infection by this very thorough removal must be diminished in a way they could never have been by the less complete and more slowly healing operation. The method employed by Barker may well form the subject of another paper.

And, finally, let me call attention to the fact so often observed, that when we come to excise the joint large sequestra are found, either the upper epiphysis or a sequestrum buried in the neck of the great trochanter, and surely in these cases excision, which is practically sequestrotomy, is urgently demanded. How can we tell that any given case advanced to suppuration has not one of these sequestra. They certainly are not rare. Quite lately we have seen a case in which an excision was nothing else than a sequestrotomy of the upper epiphysis. The wound healed at once (the neck was covered with granulations at the time of the excision), and the patient was very quickly discharged in a greatly improved condition, although there were signs that the disease was not quite at an end.

Again, in cases where there is an intra pelvic abscess with perforation through the acetabulum, it is desirable to remove the head and trochanter to give room for the drainage of the abscess.

